# Synergistic effect of venetoclax and ibrutinib on ibrutinib-resistant ABC-type DLBCL cells

**DOI:** 10.1590/1414-431X2024e13278

**Published:** 2024-10-07

**Authors:** Fengbo Jin, Limei He, Yingying Chen, Wanlu Tian, Lixia Liu, Ling Ge, Wei Qian, Leiming Xia, Mingzhen Yang

**Affiliations:** 1 Department of Hematopathology, The First Affiliated Hospital of Anhui Medical University, Hefei, Anhui, China; 2 Anhui Public Health Clinical Center, Hefei, Anhui, China

**Keywords:** Venetoclax, Ibrutinib, Ibrutinib-resistant, ABC-type DLBCL cells, Synergistic effect

## Abstract

Despite the widespread use of R-CHOP therapy in diffuse large B-cell lymphoma (DLBCL), the therapeutic efficacy for this disease remains suboptimal, primarily due to the heterogeneity of refractory and/or relapsed diseases. To address this challenge, optimization of DLBCL treatment regimens has focused on the strategy of combining an additional drug “X” with R-CHOP to enhance efficacy. However, the failure of R-CHOP combined with the BTK inhibitor ibrutinib in treating ABC-type DLBCL patients has raised significant concerns regarding ibrutinib resistance. While some studies suggest that venetoclax may synergize with ibrutinib to kill ibrutinib-resistant cells, the underlying mechanisms remain unclear. Our study aimed to validate the enhanced tumor-suppressive effect of combining ibrutinib with venetoclax against ibrutinib-resistant cells and elucidate its potential mechanisms. Our experimental results demonstrated that ibrutinib-resistant cells exhibited significant cytotoxicity to the combination therapy of ibrutinib and venetoclax, inducing cell apoptosis through activation of the mitochondrial pathway and inhibition of aerobic respiration. Furthermore, we validated the inhibitory effect of this combination therapy on tumor growth in *in vivo* models. Therefore, our study proposes that the combination therapy of ibrutinib and venetoclax is a promising treatment strategy that can be applied in clinical practice for ABC-type DLBCL, offering a new solution to overcome the urgent challenge of ibrutinib resistance.

## Introduction

Diffuse large B-cell lymphoma (DLBCL) is the most prevalent sub-type of lymphoma and a significant contributor to both incidence and mortality rates (1). R-CHOP is the current standard treatment for DLBCL, but its efficacy remains unsatisfactory due to the heterogeneity of refractory and/or relapsing disease (2,3). To address this challenge, an additional drug, referred to as “X”, is added to the R-CHOP backbone in an optimal treatment strategy to improve efficacy (4). Given the crucial role of the B-cell receptor (BCR) and its downstream BCR tyrosine kinases in malignancies arising from B cells (5,6), the failure of the combination of R-CHOP with the BTK inhibitor ibrutinib to significantly improve overall survival in ABC DLBCL patients has brought the pressing issue of ibrutinib resistance to the forefront (7). A recent study suggests that the combination of ibrutinib with venetoclax demonstrates synergistic inhibitory effects on ibrutinib-resistant cells, promoting apoptosis (8). Clinical evidence further supports the favorable efficacy and tolerability of ibrutinib in combination with venetoclax for refractory non-GCB DLBCL (9). However, the precise mechanism underlying the synergistic cytotoxicity of venetoclax with ibrutinib against ibrutinib-resistant cells remains elusive.

Understanding the development of resistance to ibrutinib is crucial to identifying effective therapeutic strategies for treating cancers such as DLBCL, which can help to overcome this challenge. Ibrutinib resistance can arise from mutations in BTK, CD79A, and CD79B (10,11), the substrate of BTK phospholipase C gamma 2 (PLCG2) (12), MYD88 (13), and CARD11 (14), as well as the upregulation of druggable survival pathways. Additionally, clonal evolution due to other genetic alterations (15) and aberrant expression of micro-RNAs (miRNAs) (16,17) can contribute to resistance mechanisms. Rational therapeutic combinations of targeted agents that inhibit adaptive pathways promoting drug resistance may help overcome these mechanisms. Several studies have reported the involvement of aberrant expression of miRNAs in the development of chemo-sensitivity or -resistance in various cancers, including DLBCL.

Venetoclax is a promising treatment option for various hematologic malignancies (18,19), including DLBCL (19). High levels of BCL2 expression, found in approximately 50% of DLBCL tissue samples, are significantly associated with poorer overall survival rate (P=0.019) and event-free survival rate (P=0.022) (6). Therefore, the molecular characteristics make venetoclax a promising candidate for treating DLBCL, and recent studies have demonstrated its effectiveness in combination with R-CHOP. The phase II CAVALLI trial demonstrated that combining venetoclax with R-CHOP resulted in a satisfactory 2-year progression-free survival rate, with comparable toxic death rates to those observed in the matched R-CHOP controls from the GOYA study (20). These findings suggest a synergistic effect when venetoclax is combined with ibrutinib in patients with the ABC subtype DLBCL. This hypothesis is supported by the rationale that combining venetoclax with ibrutinib, which targets a distinct pathway within the cell, could lead to enhanced synergistic efficacy in ABC subtype DLBCL patients, potentially having a more substantial impact on tumor growth and progression.

Together, we collaborated on a study to investigate the efficacy of the combination of venetoclax and ibrutinib in treating the ABC subtype of DLBCL, while also exploring the underlying mechanisms involved.

## Material and Methods

### Reagents

Venetoclax (ABT-199, #HY-15531), Ibrutinib (#HY-10997), and NAC (#HY-B0215) were obtained from MedChemExpress LLC (USA). All antibodies were purchased from Epitomics (USA).

### Animals

Balb/c-Nu mice (3-5 weeks old, female) were purchased from Nanjing Institute of Model Zoology (China). All experimental mice were housed in a temperature- and humidity-controlled SPF-level laminar flow rack at the Provincial Experimental Animal Center of Anhui Medical University. The animal experiments conducted were in accordance with the guidelines approved by the Institutional Animal Care and Use Committee at the University Laboratory Animal Research of Anhui Medical University.

### Cell culture

Human B-cell lymphoma cell lines HBL1 were purchased from the Shanghai Institute of Cell Biology, Chinese Academy of Sciences (China). HBL1 cells were cultured in DMEM (Thermo-Fisher, China) supplemented with 10% fetal bovine serum and 50 units/mL penicillin/streptomycin (Invitrogen, USA). The HBL1 cell lines were cultured in a humidified chamber at 37°C containing 5% CO_2_. To create ibrutinib-resistant cells (HBL-IR), HBL1 were continuously exposed to increasing concentrations of ibrutinib over several weeks, beginning with a sub-lethal dose. The ibrutinib-resistant cells were selected and maintained by culturing them in the presence of at least 1 μM ibrutinib, which ensured continued selection pressure.

### Cell viability

Cells were treated with indicated agents in 96-well tissue culture plates in DMEM supplemented with 10% FBS for 1 day. Before assessing viability using the microplate reader (Thermo Fisher Scientific, USA), the cells were treated with venetoclax, with or without ibrutinib, and monitored for proliferation using the MTT assay. To represent cell viability and combination index (CI), the average value of triplicate experiments was calculated for each dose. NAC (N-acetylcysteine) is a compound containing a mercapto group that promotes the biosynthesis of glutathione and is a common antioxidant. In this study, NAC was used to inhibit cellular oxidative stress response. HBL1-IR cells were pretreated with 10 mM NAC for 1 h and were then treated with venetoclax and/or ibrutinib for 24 h.

### CI calculation

The synergy of both drugs in killing HBL1-IR cells was evaluated by calculating the CI using CompuSyn software version 1.0 (ComboSyn Inc., USA). Synergism was indicated when the calculated CI was less than 1, additivity when the CI was equal to 1, and antagonism when the CI was greater than 1.

### Flow cytometry for apoptosis analysis

The quantification of the apoptotic cell population was carried out using the annexin V/PI double staining apoptosis kit (BD, USA) in accordance with the manufacturer's guidelines. Briefly, cells were treated with venetoclax and/or ibrutinib for 24 h, then digested, and collected for staining. Subsequently, annexin-V and PI were added and incubated for 15 min, and the apoptotic cells were acquired using a FACScan flow cytometer (BD FACSCalibur, USA). The data obtained were analyzed with FlowJo Software version VX (Tree Star, Inc., USA).

### Western blot assay

To extract the total cellular proteins, the cells were lysed using RIPA lysis buffer (#HY-K1001, MedChemExpress) supplemented with 1×protease/phosphatase inhibitor (#78441, Thermo Fisher Scientific Inc.). The collected proteins were separated by SDS-polyacrylamide gel electrophoresis and transferred onto a polyvinylidene difluoride (PVDF) membrane. The membrane was blocked with bovine serum albumin for 1 h. Primary antibodies against GAPDH (#ab9485, Abcam, USA), β-actin (#ab179467 Abcam), cleaved caspase-3 (#9661s, Cell Signaling Technology, USA), cleaved caspase-9 (#9505s, Cell Signaling Technology), cleaved PARP (#5625s, Cell Signaling Technology), BCL2 (#14093, Cell Signaling Technology), BAX (#41162, Cell Signaling Technology), BAD (#9268, Cell Signaling Technology), and cytochrome c (#4280, Cell Signaling Technology) were then incubated overnight at 4°C on a shaker, followed by treatment with horseradish peroxidase-conjugated anti-rabbit secondary antibodies (#ab6759, Abcam) for 1 h at room temperature with gentle shaking. Protein bands were visualized using ECL reagents from Pierce Biotechnology (USA) and imaged with a ChemiDoc MP system from Bio-Rad Laboratories, Inc. (USA). Band intensities were quantified using ImageJ software (NIH, USA).

### Seahorse assay

The measurement of cellular oxygen consumption rate (OCR) was performed using the Seahorse XFe24 analyzer (Agilent Technologies, USA). Briefly, HBL1-IR cells (3 replicates) were plated onto XFe24 cell culture microplates (#100777-004, Agilent Technologies) at a cell density of approximately 90% (2.5×10^4^ cells/well) with DMEM containing 10% FBS, and incubated at 37°C with 5% CO_2_ humidity until complete adhesion. Prior to the experiment, the culture medium was replaced with 500 μL Seahorse XF base medium (#103575-100, Agilent Technologies) and supplemented with reagents for mitochondrial oxidative metabolism analysis. HBL1-IR were then incubated at 37°C for 1 h in a CO_2_-free incubator. A cartridge containing oxygen- and pH-sensitive probes was pre-treated overnight with calibration solution (#103059-000, Agilent Technologies) at 37°C.

The assessments of OCR and extracellular acidification rate (ECAR) were conducted during the time course before and after injection of the following compounds. OCR was measured (#103015-100, Agilent Technologies) in response to the introduction of the following compounds: 1 mM oligomycin, 0.5 mM FCCP [carbonyl cyanide] (trifluoromethoxy) phenylhydrazone, and 0.5 mM antimycin A.

In accordance with the ECAR measurement protocol (#103020-100, Agilent Technologies), the following compounds were administered: i) 10 mM glucose, ii) 1 μM oligomycin, and iii) 50 mM 2-deoxyglucose (2-DG). Normalization of ECAR and OCR values to total protein per well was performed, with the resulting data points reflecting the average values during the measurement period. These values are reported as absolute rates (mpH/min/μg for ECAR and pmol/min/μg for OCR).

### *In vivo* xenograft study

Female nude mice aged 6-8 weeks were obtained from Anhui Medical University and bred under specific pathogen-free conditions. The mice were subcutaneously injected with 5×10^6^ HBL-IR cells in a volume of 100 µL into the right hind legs. When the tumors reached a volume of 100 mm^3^, the mice were randomly assigned to different treatment groups: Group I (Control, PBS); Group II (ibrutinib, 12 mg/kg once daily, via intragastric administration); Group III (venetoclax, 40 mg/kg once daily, via intragastric administration); and Group IV (ibrutinib and venetoclax combination, 12 and 40 mg/kg, respectively, via oral gavage once daily). The tumor volumes were calculated as tumor volume = 1/2 × L (length) × W^2^ (width). For the tumor models, the tumors were measured 2-3 times per week, and the mice were euthanized when the tumors reached humane endpoints (when the longest diameter of the tumor reached or exceeded 2 cm). Daily animal care and husbandry were provided by the Experimental Animal Center of Anhui Medical University. The study adhered to the guidelines for the care and use of laboratory animals as set by Anhui Medical University, and the animal experimental protocols were approved by the Animal Care and Use Committee of Anhui Medical University.

### Statistical analysis

GraphPad Prism 9 (GraphPad Software, USA) was used to analyze all data. Student’s *t*-test and one-way ANOVA (followed by Tukey's test) were used and variables are reported as means±SD in the figures. A P value less than 0.05 indicates statistical significance.

## Results

### Venetoclax and ibrutinib monotherapy and combination treatment inhibited proliferation of HBL-IR cells

**Figure 1:**
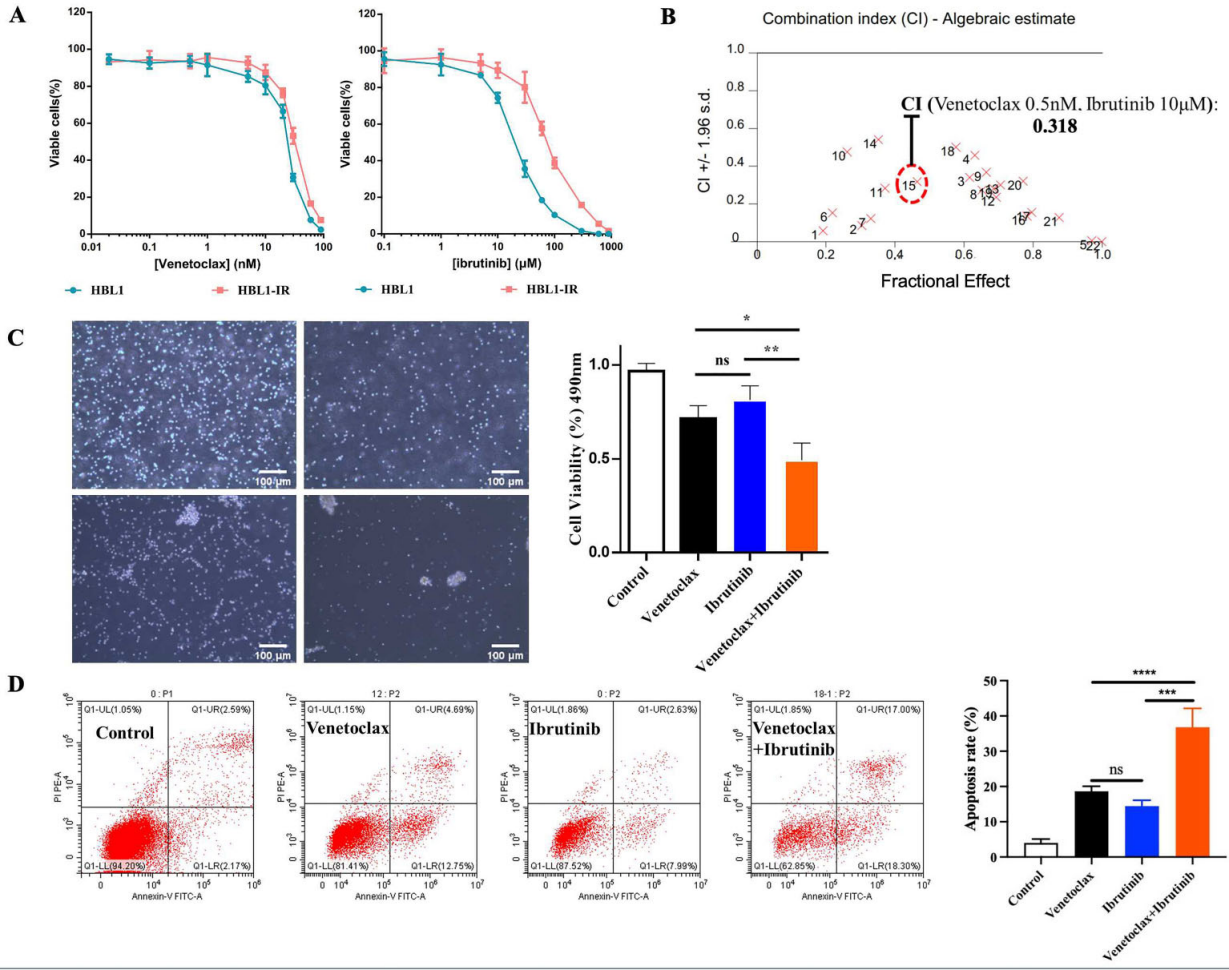
**A**, Viability and half maximal inhibitory concentration (IC50). **B**, Combination index analysis results. **C**, Synergistic inhibitory effects shown through morphology analysis (scale bar 100 μm). **D**, Flow cytometry analysis of cell death using annexin-V staining. The percentages of cells undergoing apoptosis in the control, venetoclax, ibrutinib, and combination groups were 4.033±1.069%, 18.63±1.45%, 14.43±1.68%, and 36.80±5.336%, respectively. Data are reported as means±SD. *P<0.05, **P<0.01, ***P<0.001, ****P<0.0001 (ANOVA). ns: non-significant.

The ibrutinib-resistant HBL-IR cells were obtained through the *in vitro* culture of parental cell lines with gradually escalating concentrations of ibrutinib. The half maximal inhibitory concentration (IC50) of ibrutinib-resistant HBL-IR cells was 76.50 μmol/L, compared to 22.60 μmol/L for parental cells; this fold change exceeding 3 indicates the development of significant resistance (Figure 1A). Venetoclax at concentrations ranging from 0.01 to 10 nM showed less than 20% inhibition rate on HBL1 and HBL1-IR cells. CI analysis (Table 1) of the effect of venetoclax in combination with ibrutinib at different concentrations on ibrutinib-resistant HBL1-IR cells revealed a CI of 0.318 (Figure 1B). Despite the relatively modest cytotoxicity observed with the individual treatments of 10 μM ibrutinib and 0.5 nM venetoclax in HBL1-IR cells, they manifested a noteworthy synergistic capability. Consequently, these specific concentrations were retained for subsequent studies. Microscopic scrutiny of the morphology of HBL1-IR cells further substantiated the synergistic inhibitory effects (Figure 1C). Individual treatments with 10 μM ibrutinib and 0.5 nM venetoclax caused a discernible reduction in cell density compared to the Control. However, the combined treatment group, which exhibited a more pronounced inhibitory effect on cells, was deemed to effectively showcase the synergistic cytotoxicity.

**Table 1:** Values of the combination index for ibrutinib and venetoclax in the *in vitro* experiment from the points shown in Figure 1B. Bold type indicates peak synergistic effect.

Ibrutinib (μM)	Venetoclax (nM)	Marker
0.1	0.1	1
0.1	0.5	2
0.1	20	3
0.1	30	4
0.1	60	5
1	0.1	6
1	0.5	7
1	20	8
1	30	9
5	0.1	10
5	0.5	11
5	20	12
5	30	13
10	0.1	14
**10**	**0.5**	**15**
10	20	16
10	30	17
30	0.1	18
30	0.5	19
60	0.1	20
60	0.5	21
100	0.1	22

To further investigate the effects of venetoclax and ibrutinib on HBL-IR cells, we evaluated their ability to trigger cell death. Our results showed that the percentage of cells undergoing cell death in the control, venetoclax, ibrutinib, and combination groups were 4.033±1.069%, 18.63±1.45%, 14.43±1.68%, and 36.80±5.336%, respectively. These findings suggested that the combination treatment led to a significantly higher increase in cell death compared to the substances alone (Figure 1D).

### Venetoclax activated the caspase-dependent mitochondrial apoptotic pathway, increasing the sensitivity of HBL1-IR cells to apoptosis induced by ibrutinib

**Figure 2:**
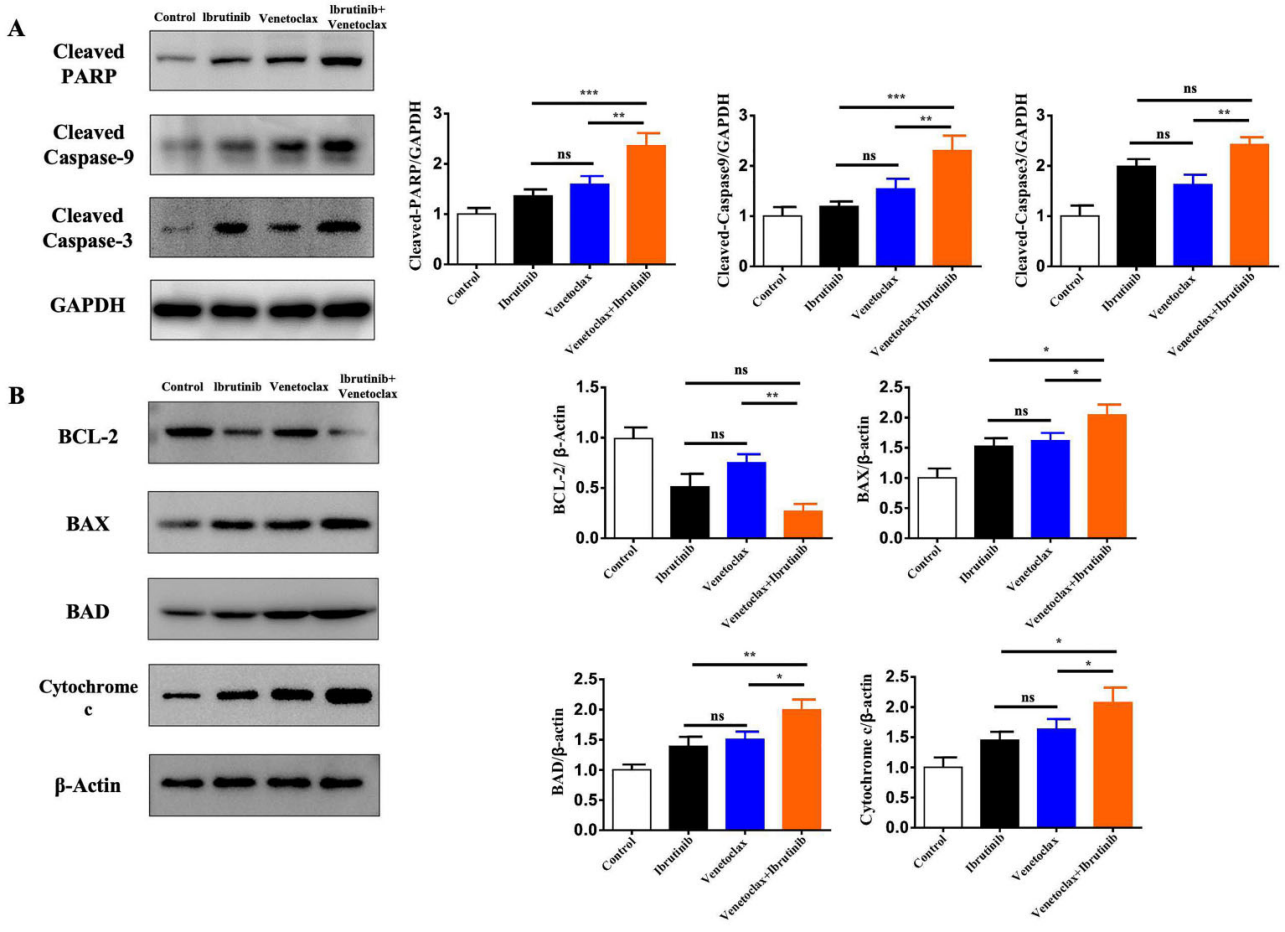
The expression of apoptosis-related proteins was evaluated in HBL1-IR cells treated with venetoclax, ibrutinib, or their combination. **A**, The expressions of cleaved-PARP, cleaved-caspase 9, and cleaved-caspase 3 were detected in the HBL1-IR cell line subjected to monotherapy and combination therapy of venetoclax and ibrutinib. **B**, Mitochondrial apoptosis-related proteins were detected in HBL1-IR cells treated with venetoclax, ibrutinib, or their combination. Each experiment was repeated three times. Data are reported as means±SD. *P<0.05, **P<0.01, ***P<0.001 (ANOVA). ns: non-significant.

Our results of mitochondria-mediated apoptotic pathway proteins demonstrated that the combination therapy of venetoclax and ibrutinib significantly increased the expression of cleaved caspase-3/9 and cleaved PARP compared to treatment with the substances alone (P<0.05, Figure 2A). There were no significant differences in the protein levels of BCL2, BAX, BAD, and cytochrome c between the groups treated with the substances alone in HBL1-IR cells (P>0.05). However, in the combination group, the protein levels of BCL2, Bax, BAD, and cytochrome c were significantly different compared to the venetoclax and ibrutinib monotherapy groups (P<0.05, Figure 2B).

### Effect of venetoclax in combination with ibrutinib treatment on the oxygen consumption levels of HBL1-IR cells

**Figure 3:**
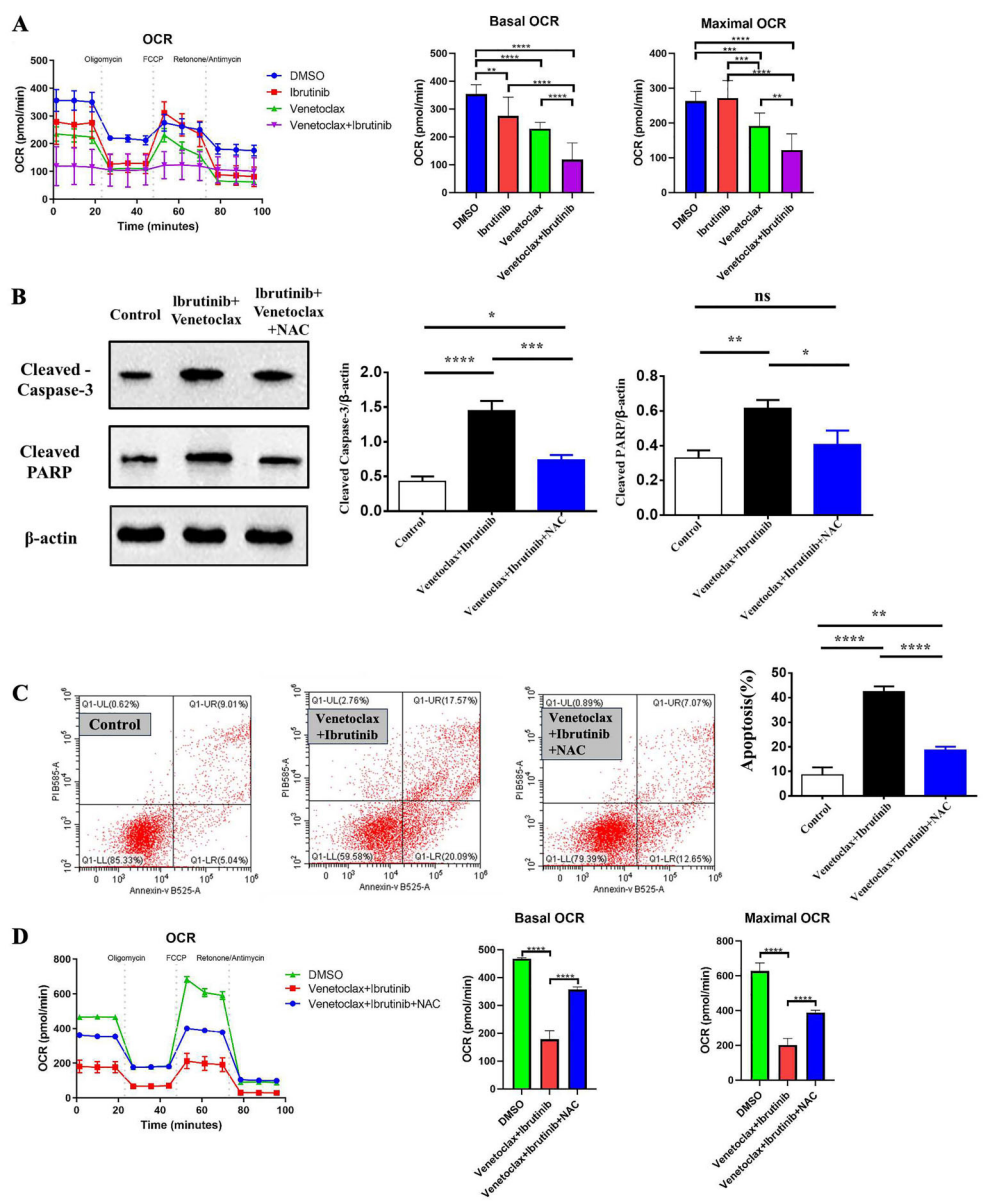
The effect of venetoclax and ibrutinib treatment on the oxygen consumption rate (OCR) of HBL1-IR cells. **A**, *In vitro* aerobic oxidation levels of HBL1-IR cells treated with venetoclax, ibrutinib, or their combination at selected concentrations. **B**, Effect of N-acetylcysteine (NAC) on expression of cleaved PARP and caspase 3 proteins of HBL1-IR cells treated with venetoclax and ibrutinib in combination. **C**, Results of flow cytometry analysis showing apoptosis levels of HBL-IR cells treated with venetoclax and ibrutinib in combination. **D**, Effect of NAC on aerobic oxidation. Each experiment was repeated three times. Data are reported as means±SD. *P<0.05, **P<0.01, ***P<0.001, ****P<0.0001 (ANOVA). ns: non-significant.

We found that there was no significant difference (P>0.05) in OCR among the groups treated with ibrutinib alone in HBL1-IR cells, but there was a difference in the basal OCR level compared to the control group (P<0.05). Treatment with venetoclax resulted in a significant difference (P<0.05) in both basal and maximum OCR levels compared to the control group in HBL1-IR cells. In the combination group, there was a significant statistical difference (P<0.05, Figure 3A) in both basal and maximum oxygen consumption rates compared to the venetoclax and ibrutinib monotherapy groups.

We measured the protein levels of active cleaved PARP and caspase 3, which are apoptosis-related proteins. We found that after NAC blocked oxidative stress, the protein levels of active cleaved PARP and caspase 3 in the cleaved bodies of the cells were significantly decreased compared to the venetoclax and ibrutinib combination treatment group, and there was no significant difference compared to the control group (P>0.05, as shown in Figure 3B). Furthermore, flow cytometry analysis showed that blocking cellular oxidative stress with NAC resulted in a significant decrease in apoptosis levels of HBL-IR cells treated with the combination treatment (Figure 3C). In addition, blocking the oxidative stress level with NAC restored the aerobic oxidation level of HBL-IR cells compared to the combined treatment (as seen in Figure 3D).

### Combination treatment of venetoclax and ibrutinib inhibited tumor growth in the xenograft model

**Figure 4:**
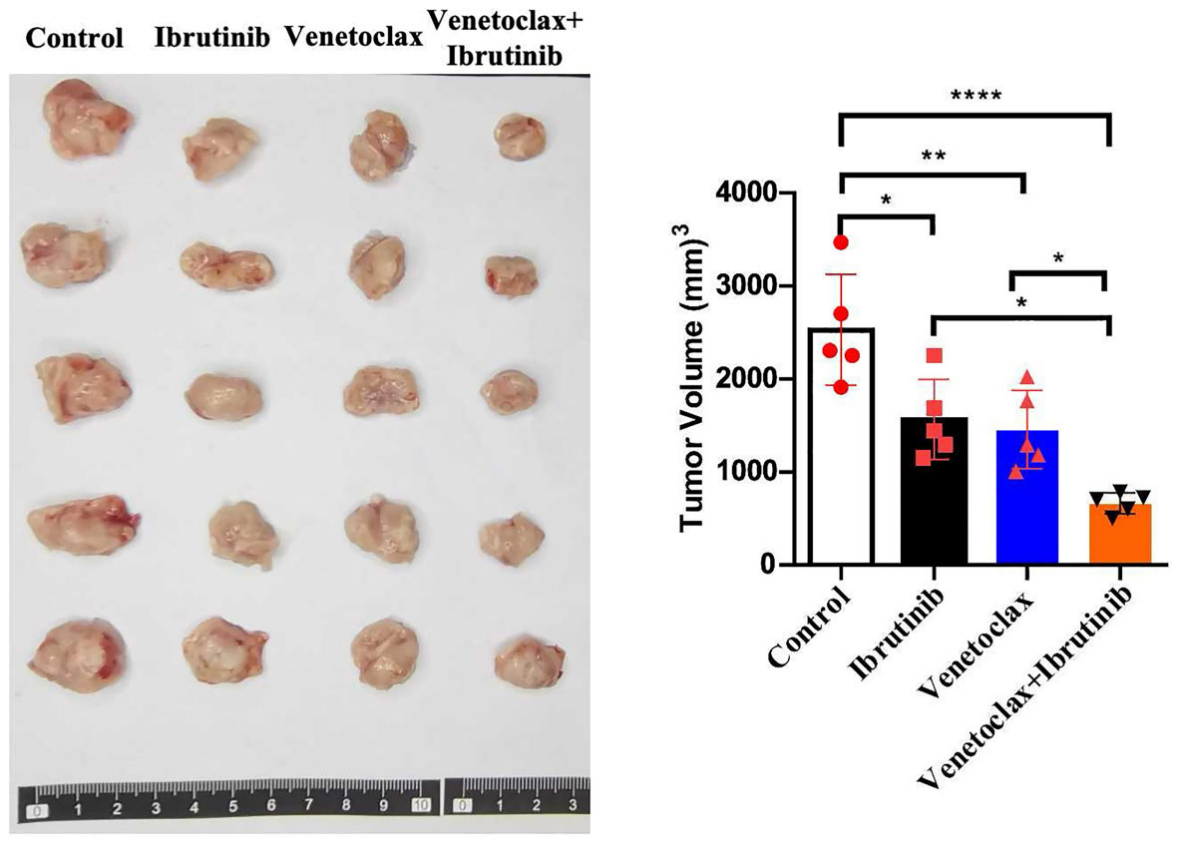
Tumor size and volume of HBL1-IR cell line tumor-bearing mice, with each group consisting of five mice treated with either venetoclax or ibrutinib or their combination. Data are reported as means±SD. *P<0.05, **P<0.01, ****P<0.0001 (ANOVA).

Our findings of the *in vivo* experiment indicated that both venetoclax and ibrutinib monotherapy was effective in reducing tumor growth compared to the control group. Furthermore, the combination therapy induced a more significant reduction (P<0.0001) in tumor growth, as evidenced by reduced tumor volume (Figure 4).

## Discussion

Non-Hodgkin lymphomas, a common type of hematopoietic cancer, is witnessing a rising incidence and mortality rates. Currently, the standard first-line treatment for this disease is R-CHOP (21). However, there is significant variability in the response to R-CHOP among patients with DLBCL. Studies have shown that the 2-year progression-free survival (PFS) for ABC-DLBCL is only 28%, whereas for GCB-DLBCL it is as high as 64%, with 2-year overall survival (OS) rates being 46 and 78%, respectively (22). These results highlight the insensitivity of ABC DLBCL to R-CHOP and underscore the importance of optimizing treatment strategies for this patient population.

Regrettably, the combination of lenalidomide or ibrutinib with R-CHOP has not significantly improved the efficacy in treating ABC-DLBCL (23,24). Mechanistically, ABC-DLBCL tumors exhibit a substantial amount of genetic variability, with distinct subgroups identified by genetic mutations showing significant differences in 5-year survival rates (23,25). Overall, compared to Germinal Center B-cell-like (GCB) DLBCL, ABC-DLBCL is more reliant on the B-cell receptor signaling pathway (23,25,26). Furthermore, ABC subtype DLBCL tumor cells exhibit specific gene mutations (27), including MYD88 (20%), CD79a/b (20%), CARD11 (10%), MALT1, BCL10, TNFAIP3, and MYD8. These mutations impact pathways like B-cell receptor and Toll-like receptor signaling, which in turn lead to the activation of the NF-kB pathway. We hypothesized that by combining ibrutinib with targeted BCL2 therapies together with the existing R-CHOP+X regimen, we may enhance its anti-tumor effect and overall efficacy.

BCL2 is a mitochondrial membrane protein that regulates the apoptotic pathway of cells. Venetoclax is a selective inhibitor of the BCL2 protein and can inhibit the release of cytochrome C. The activity balance between BCL2 superfamily proteins and BH3 family proteins determines the biological characteristics of cells (28,29). Venetoclax is a small molecule drug that selectively inhibits the BCL2 protein by mimicking BH3 proteins. This drug can bind to the hydrophobic pocket area of the BCL2 protein with high affinity and competitively bind to other BH3 structures targeting proteins (18). Experiments have shown that venetoclax can inhibit the binding of BCL2 superfamily proteins and BH3 proteins and reduce the release of mitochondrial cytochrome C (30).

In our study, combining venetoclax, which targets BCL2, with ibrutinib in an X regimen, we observed a synergistic effect on HBL1-IR cells, an ibrutinib-resistant variant of the ABC-type DLBCL cell line HBL1. The synergistic effect peaked with a combination of 10 μM venetoclax and 0.5 nM ibrutinib, surpassing the efficacy of either drug alone. Microscopic examination showed a marked reduction in cell survival in treated HBL1-IR cells, with the combination therapy leading to the most significant decrease. Apoptosis levels were elevated in both single-agent treatments, but the combination therapy increased the ratio of early and late apoptotic cells to over 30%. Western blotting confirmed increased levels of cleaved PARP, caspase 9, and caspase 3 in the combination group, suggesting a potentiated induction of apoptosis. These findings indicated that the venetoclax and ibrutinib combination significantly enhanced apoptosis in HBL1-IR cells, offering a promising therapeutic strategy against ibrutinib resistance.

We found that venetoclax combined with ibrutinib primarily targeted ibrutinib-resistant HBL1-IR cells through mitochondrial apoptosis induction and inhibition of the mitochondrial oxidative respiratory chain. With post-treatment with venetoclax, BCL2 levels in HBL1-IR cells were unchanged, but there was a notable increase in pro-apoptotic proteins like BAD and Bax and a significant rise in cytochrome C release. Ibrutinib alone decreased BCL2 and increased BAD and Bax. Notably, the venetoclax-ibrutinib combination further elevated BAD and Bax, reduced BCL2, and markedly enhanced cytochrome C release, suggesting venetoclax's role in promoting apoptosis in resistant ABC-DLBCL cells. OCR measurements showed that venetoclax significantly lowered basal and maximal OCR in HBL1-IR cells, inhibiting their aerobic respiration. Ibrutinib also reduced basal OCR. The combination treatment further decreased both OCR values, indicating impaired mitochondrial function and suggesting that venetoclax and ibrutinib synergize to disrupt mitochondrial integrity and respiration, contributing to the elimination of ibrutinib-resistant HBL1-IR cells.

The mitochondrial apoptosis pathway is often triggered by stress signals like oxidative stress, endoplasmic reticulum stress, and DNA damage, with oxidative stress being a key activator (31). In our study, we utilized NAC to assess if the combination of venetoclax and ibrutinib induced oxidative stress-mediated mitochondrial apoptosis in HBL1-IR cells. We observed that NAC significantly reduced the cleavage of apoptosis-related proteins such as caspase3 and PARP, which are induced by the venetoclax-ibrutinib combination, indicating suppressed apoptosis. Additionally, OCR measurements revealed that NAC could counteract the effects on apoptosis-related proteins caused by venetoclax and ibrutinib, for both basal and maximal OCR levels. These findings implied that venetoclax, in synergy with ibrutinib, enhancde intracellular reactive oxygen species production, activated the mitochondrial apoptosis pathway, and impeded cellular aerobic respiration.

Finally, we verified the effectiveness of venetoclax combined with ibrutinib against ibrutinib-resistant HBL1-IR cell tumor-bearing mice *in vivo*. We found that the tumor volume of tumor-bearing mice treated with venetoclax or ibrutinib alone was significantly reduced compared to the control group, and the tumor volume of tumor-bearing mice treated with venetoclax combined with ibrutinib was significantly smaller than that of the monotherapy group, further demonstrating the synergistic killing effect of venetoclax combined with ibrutinib on ibrutinib-resistant cell tumors.

In conclusion, venetoclax combined with ibrutinib proved to be a promising X drug for the treatment of ABC-type DLBCL in clinical practice.
